# Enhancement of sleep slow waves: underlying mechanisms and practical consequences

**DOI:** 10.3389/fnsys.2014.00208

**Published:** 2014-10-28

**Authors:** Michele Bellesi, Brady A. Riedner, Gary N. Garcia-Molina, Chiara Cirelli, Giulio Tononi

**Affiliations:** ^1^Department of Psychiatry, University of Wisconsin-MadisonMadison, WI, USA; ^2^Clinical Sites Research Program, Philips Group InnovationBriarcliff, NY, USA

**Keywords:** EEG, acoustic stimulation, arousal systems, closed-loop, NREM sleep

## Abstract

Even modest sleep restriction, especially the loss of sleep slow wave activity (SWA), is invariably associated with slower electroencephalogram (EEG) activity during wake, the occurrence of local sleep in an otherwise awake brain, and impaired performance due to cognitive and memory deficits. Recent studies not only confirm the beneficial role of sleep in memory consolidation, but also point to a specific role for sleep slow waves. Thus, the implementation of methods to enhance sleep slow waves without unwanted arousals or lightening of sleep could have significant practical implications. Here we first review the evidence that it is possible to enhance sleep slow waves in humans using transcranial direct-current stimulation (tDCS) and transcranial magnetic stimulation. Since these methods are currently impractical and their safety is questionable, especially for chronic long-term exposure, we then discuss novel data suggesting that it is possible to enhance slow waves using sensory stimuli. We consider the physiology of the K-complex (KC), a peripheral evoked slow wave, and show that, among different sensory modalities, acoustic stimulation is the most effective in increasing the magnitude of slow waves, likely through the activation of non-lemniscal ascending pathways to the thalamo-cortical system. In addition, we discuss how intensity and frequency of the acoustic stimuli, as well as exact timing and pattern of stimulation, affect sleep enhancement. Finally, we discuss automated algorithms that read the EEG and, in real-time, adjust the stimulation parameters in a closed-loop manner to obtain an increase in sleep slow waves and avoid undesirable arousals. In conclusion, while discussing the mechanisms that underlie the generation of sleep slow waves, we review the converging evidence showing that acoustic stimulation is safe and represents an ideal tool for slow wave sleep (SWS) enhancement.

Sleep is thought to be a universal phenomenon. Despite representing a behavioral state of almost total disconnection from the environment and, therefore, being inherently dangerous, sleep has been identified in every species carefully studied so far (Tobler, 2005; Cirelli and Tononi, 2008; Tononi and Cirelli, 2012). It is unknown when and why sleep emerged in evolution, but the simplest hypothesis is that sleep evolved to serve at least one core function in all species (Cirelli and Tononi, 2008).

Sleep is a tightly regulated homeostatic process. A sleep deficit elicits a compensatory increase in the intensity and duration of sleep, while excessive sleep reduces sleep propensity (Borbély and Achermann, 1999, 2000, 2005; Cirelli and Tononi, 2008). In addition, the quality of wake impacts the intensity of subsequent sleep; in both rodents and humans, intensive learning involving a specific brain region induced a local increase of slow wave activity (SWA, the EEG power between 0.5 and 4 Hz during non-rapid eye movement, NREM sleep) in the very same region during the following sleep, suggesting an experience-dependent local regulation of sleep (Huber et al., 2004, 2006; Vyazovskiy and Tobler, 2008; Hanlon et al., 2009; Krueger and Tononi, 2011; Hung et al., 2013).

It has been proposed that sleep is needed to reestablish “synaptic homeostasis”, which is challenged by the remarkable plasticity of the brain (Tononi and Cirelli, 2003, 2006, 2014). Plastic processes occurring during wakefulness result in a net increase in synaptic strength in many brain circuits. Increased synaptic strength has various costs at the cellular and systems level including higher energy consumption (Attwell and Laughlin, 2001), greater need of cellular supplies to synapses leading to cellular stress (Kuhl et al., 1992; Li et al., 2004), and associated changes in glial cells (Reichenbach et al., 2010). Increased synaptic strength also reduces the selectivity of neuronal responses (Balduzzi and Tononi, 2013) and saturates the ability to learn, as suggested by electrophysiological evidence in neocortex and hippocampus (Foster et al., 1996; Rioult-Pedotti et al., 1998, 2000; Whitlock et al., 2006). By renormalizing synaptic strength, sleep could therefore restore the brain to a baseline condition (Tononi and Cirelli, 2003, 2006, 2014). Several lines of evidence have also shown that lack of sleep leads unavoidably to negative consequences for the organism (Montagna and Lugaresi, 2002; Rechtschaffen and Bergmann, 2002; Shaw et al., 2002; Patel and Hu, 2008; Van Cauter et al., 2008). In humans, even modest sleep restriction leads to cognitive impairment, decreased work productivity, mortality related to automobile crashes, and other adverse events likely related to intrusion of sleep into waking (Banks and Dinges, 2007). These short-lasting sleep-like events, known as behavioral microsleeps, can manifest as a complete failure to respond during an active task, slow eye movements, cessation of blinking and/or head nods, and are usually accompanied by an increase in theta activity in the waking electroencephalogram (EEG; Priest et al., 2001; Blaivas et al., 2007). In addition to “global” microsleeps, sleep deprivation can lead to the occurrence of local sleep-like activity (OFF periods in neuronal firing, typically associated with a local theta or slow wave) in an otherwise awake brain, and cause specific, intermittent performance impairments (Vyazovskiy et al., 2011). This “local sleep” phenomenon was initially demonstrated in rats, but it was recently described also in humans (Hung et al., 2013).

## Slow waves and their importance in sleep

The best characterized marker of the homeostatic regulation of sleep are the slow waves of NREM sleep (Achermann and Borbély, 2003). They are the most prominent EEG event during sleep; they appear as spontaneous large oscillations of the EEG signal occurring approximately once every second in the deepest stage of NREM sleep. Each oscillation consists of an up state, in which neurons fire irregularly at frequencies typical of waking or higher, followed by a hyperpolarized phase, where neurons cease to fire (down state) (Steriade et al., 1993b, 2001; Amzica and Steriade, 1998b; Destexhe et al., 1999).

A compelling feature of slow waves is that they are homeostatically regulated. In general, the longer one has been awake, the more frequent and larger are the slow waves during the subsequent sleep. Therefore, SWA is used as an index of sleep need. Sleep SWA is high in early sleep, when sleep pressure is physiologically elevated, and decreases progressively to reach low levels in late sleep (Tobler and Borbély, 1986; Franken et al., 1991; Vyazovskiy et al., 2007). Moreover, sleep SWA increases further after sleep deprivation, and is reduced by naps (Borbély and Achermann, 2000; Tobler, 2005).

Recently, it has been proposed that, in adults, the homeostatic decline of SWA during sleep is due to a progressive decrease in synaptic strength, which is thought to increase during wakefulness and decrease during sleep (Tononi and Cirelli, 2003, 2006, 2014). There is also some evidence that slow waves may reflect not only the regulation of synaptic strength, but also have a direct causal role in mediating synaptic renormalization during sleep. Experiments performed in rodents and computer simulations have shown that the alternate periods of discharge and silence of neurons, which characterize the behavior of thalamo-cortical neurons during the slow waves, are ideally suited to induce synaptic depression (Kemp and Bashir, 2001; Lubenov and Siapas, 2008; Lanté et al., 2011). In addition, the low levels of acetylcholine and catecholamines that exist during slow wave sleep (SWS) may facilitate the occurrence of synaptic depression (Harley, 1991; Seol et al., 2007).

According to this view, slow waves should mediate at least some of the beneficial functions of sleep on the brain (Tononi and Cirelli, 2003, 2006, 2014). Indeed, several lines of evidence support this prediction. The role of slow waves in the consolidation of memories has been investigated extensively (Marshall and Born, 2007; Diekelmann and Born, 2010; Rasch and Born, 2013). For example, when assessing post-learning changes in sleep, a local increase of SWA was observed in the very same brain areas previously activated when subjects learned implicitly to adapt their movements to a rotated display (Huber et al., 2004). Moreover, the local increase in SWA after learning correlated with improved performance in the rotation adaptation task after sleep (Huber et al., 2004). By contrast, 12 h of arm immobilization induced a reduction of performance in a motor reaching task, and a subsequent decrease of SWA during sleep over the brain regions involved in that task, thereby confirming the tight link between slow waves and learning (Huber et al., 2006). Along the same lines, it was also reported that slow waves (<1 Hz) are more synchronized following intense declarative learning (Mölle et al., 2004). Other studies demonstrated better retention of declarative memories after SWS than after a control interval filled with wakefulness (Plihal and Born, 1997), and the improvement was greater with longer sleep duration (Diekelmann et al., 2012). In addition, Wilhelm et al. (2011) found that memories expected to be retrieved were the ones that benefited the most from SWS. Perhaps the most direct demonstration about the beneficial role of slow waves comes from studies in which selective slow wave deprivation during the night was carried out in healthy subjects. This manipulation, which did not affect sleep time and efficiency, prevented the improvement in performance after visuo-motor and visuo-perceptual tasks, and the changes in performance after slow wave deprivation were correlated with SWA changes, suggesting a casual role for slow waves in the sleep-dependent improvement of cognitive performance (Aeschbach et al., 2008; Landsness et al., 2009).

In addition, other studies have reported that the beneficial role of SWS might not be limited to the brain. For instance, there is evidence that both reduction in total sleep duration with SWS largely preserved and marked reduction of SWS with preservation of total sleep duration have a negative impact on the hypothalamic-pituitary-adrenal axis, in particular on the control of glucose metabolism (Van Cauter et al., 2008; Copinschi et al., 2014). Modifications of the cortisol 24 h profile were observed after few days of sleep restriction (Guyon et al., 2014), while a marked reduction of insulin sensitivity was reported after selective suppression of SWS (Tasali et al., 2008). Similarly, the autonomic control of heart rate and body temperature can be affected by sleep loss (Vaara et al., 2009; Romeijn et al., 2012). Although the role of REM or light NREM sleep cannot be easily ruled out in these studies, it is plausible that SWS exerts a beneficial function also on peripheral systems.

## Slow wave enhancement: a possible way to improve the restorative functions of sleep

Given the pivotal role of slow waves during sleep, it is not surprising that several efforts have been made to increase sleep efficacy by potentiating SWA. Recently, a number of drugs have been shown to increase SWS. Although acting on different synaptic sites, overall the slow wave enhancing effect of these drugs is mediated by enhancing GABAergic transmission. Specifically, clinical investigations showed that both tiagabine and gaboxadol increased the duration of SWS after sleep restriction (Mathias et al., 2001; Walsh et al., 2008; Walsh, 2009; Feld et al., 2013). Tiagabine also improved performance on cognitive tasks evaluating executive functions and reduced the negative effects of sleep restriction on alertness (Walsh et al., 2006). Although these results are promising, pharmacological approaches to sleep enhancement often raise issues related to dependence and tolerance, and are commonly associated with residual daytime side effects.

To overcome these limitations, one strategy is to enhance deep sleep non-pharmacologically, by stimulating the brain with electrical currents or magnetic fields. A study by Marshall et al. (2006) used intermittent transcranial direct-current stimulation (tDCS) applied at 0.75 Hz for 5-min intervals separated by 1-min off periods after SWS onset, and found an increase in the EEG power in the slow oscillation band (<1 Hz) during the stimulation-free intervals. This increase was associated with enhanced retention of hippocampal-dependent declarative memories, suggesting a causal role for slow waves in sleep-associated memory consolidation (Marshall et al., 2006). Using a similar paradigm, Reato et al. (2013) reported an acceleration of the SWA homeostatic decay in subjects stimulated by tDCS at the beginning of SWS. However, the actual impact of tDCS on physiological sleep is hard to evaluate for several reasons. The recorded EEG during the stimulation is strongly affected by electrical artifacts, preventing a detailed EEG analysis. Furthermore, although tDCS results in sustained and widespread changes in regional neuronal activity, it produces a complex pattern of activated and deactivated brain areas, making the impact on slow waves difficult to predict (Lang et al., 2005). In another study, Massimini et al. (2007) demonstrated that slow waves can be triggered by directly perturbing the cortex during NREM sleep using trans-cranial magnetic stimulation (TMS). Unlike tDCS, the EEG could be recorded concurrently to test the direct impact of TMS. Importantly, virtually every TMS pulse, when in the appropriate location, was able to trigger a full-fledged slow wave that started under the coil and spread to the rest of the brain. However, the long-term effect of repeated exposure to either tDCS or TMS is unknown. Thus, other research has focused on the possibility of inducing slow waves in a more physiological natural manner, by exploiting sensory stimulation.

Historically, vestibular stimulation was the first to be tested as a tool to promote sleep induction, perhaps because of the long-standing notion that physical rocking of a baby, or swinging in a hammock, can be helpful in inducing sleep (Woodward et al., 1990). Indeed, studies carried out in infants demonstrated that vestibular stimulation decreased the proportion of active behavior and concomitantly increased the time spent in quiet sleep (Cordero et al., 1986). In a larger study in healthy adults, bilateral electrical stimulation of the vestibular apparatus shortened sleep onset latency in comparison to sham nights where no stimulation was provided (Krystal et al., 2010). Another recent study reported a facilitated transition from waking to sleep and an increase of SWA in subjects exposed to gentle rocking during an afternoon nap (Bayer et al., 2011). According to the authors, at least one of the mechanisms by which vestibular and, more generally, proprioceptive stimulation could promote sleep and boost SWA involves direct or indirect vestibular and somatosensory projections to the reticular formation, thalamus, and hypothalamus, which could in turn enhance synchronous activity in thalamo-cortical networks (Bayer et al., 2011). In rats, olfactory stimulation induces slow waves (Fontanini et al., 2003; Fontanini and Bower, 2006), but this method had no effect when applied to humans (Tononi et al., 2010), probably because olfactory stimuli have only modest impact on human thalamo-cortical networks (Carskadon and Herz, 2004).

**Figure 1:**
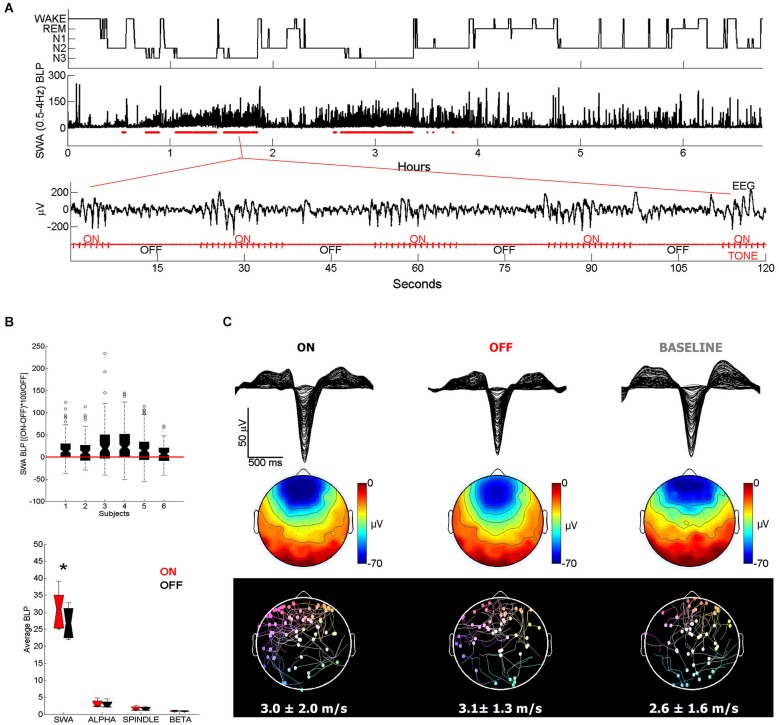
**(A)** Representative example of acoustic stimulation delivered in 15-s blocks during deeper stages of NREM sleep (N2 and N3, 50 ms tones played with an inter-tone-interval of 1 s). A custom algorithm delivered acoustic stimuli automatically, using the ongoing EEG to examine sleep and adjust tone timing and volume. Hypnogram and SWA band-limited power (BLP) for channel F3-M2 along with the stimulation blocks shown below in red (2 min of EEG and tone stimulation are expanded below). Note that during ON blocks slow waves are more numerous and larger. **(B)** All subjects (*n* = 6) showed overall increases in SWA (top plot) in ON blocks relative to the temporally adjacent OFF blocks, while other frequency ranges did not change (bottom plot). * indicates significantly different based on a paired *t*-test, Bonferroni corrected for multiple comparisons (*p* < 0.0125). **(C)** Top plot shows a butterfly plot (all channels overlaid) averaging across all 100 slow waves aligned by the negative peak. Slow waves were randomly selected for comparison from the ON and OFF periods of the stimulation night and from a BASELINE (no stimulation) night. Middle plot shows the average scalp voltage topography at the negative peak. Bottom plot shows the traveling of individual waves and their average speed below. Each dot represents the origin of the wave and the line describes its traveling. Slow waves were detected globally based on standard criteria and traveling was calculated from the negative peak lag distribution of each wave (Siclari et al., 2014).

The effect of somatosensory and auditory stimulation was assessed by Tononi et al. (2010). While the change observed with somatosensory stimulation was minor, acoustic stimulation was particularly efficacious in enhancing sleep slow waves. Specifically, using an intermittent stimulation in which tones were played in blocks of 15 s spaced out by stimulation-free intervals, slow waves appeared remarkably large and numerous during the stimulation blocks (Figure 1A; Riedner et al., 2012). Thus, when compared to the temporally adjacent stimulation free intervals, stimulation blocks displayed increases in SWA (ranging from 6 to 27%), whereas band-limited power (BLP) in the alpha (8–12 Hz), spindle (12–16 Hz), and beta (16–25 Hz) ranges did not change significantly (Figure 1B). In addition, high-density EEG studies (hdEEG, 256 channels) showed that the morphology, topography, and travelling patterns of induced slow waves were indistinguishable from those of spontaneous slow waves observed during natural sleep (Figure 1C). A recent study designed to establish the capacity to learn during sleep used acoustic tones delivered during NREM and REM sleep, and found that EEG SWA increased following tone presentation during NREM sleep (Arzi et al., 2012). In another recent study healthy young subjects were exposed to continuous acoustic stimulation at 0.8 Hz starting 2 min before lights were turned off and lasting for 90 min. Subsequent staging and EEG analysis showed an increase of slow oscillation activity (0.5–1 Hz) during the rhythmic stimulation as compared to a sham condition with no stimulation (Ngo et al., 2013a). Of note, the stimulation did not increase the number of total arousals, despite the fact that subjects took longer to fall asleep when stimulated (Ngo et al., 2013a). Lastly, the effectiveness of acoustic stimulation in enhancing slow waves was confirmed in another study in which acoustic pulses delivered during the slow wave up states increased the size of the following slow waves (<1 Hz). The stimulation also improved declarative memory performance as compared to control nights when either the stimulation was delivered out of phase (during the down states) or no stimulation was provided (Ngo et al., 2013b).

In conclusion, although other modalities of sensory stimulation (e.g., vestibular) deserve consideration, there is converging evidence that auditory stimulation is a good choice for enhancing slow waves, because it is safe, easily controllable, and can be administered non-obtrusively during sleep.

## Enhancing slow waves with acoustic stimulation

The use of acoustic stimulation in sleep and EEG research is almost as old as the fields themselves. In sleep research, acoustic stimuli are commonly used to test the change in arousal threshold as a function of sleep depth. Further studies assessed the different brain responses to auditory stimulation across the sleep/wake cycle, and others investigated the role of rhythmic stimulation on the acceleration of sleep onset (Grossman, 1949; Oswald, 1960; Zung and Wilson, 1961; Bohlin, 1971). Nonetheless, how acoustic stimulation leads to slow wave enhancement is still an open question. In the following section we offer a working hypothesis on the underlying mechanisms after reviewing the physiology of the K-complex (KC), a peripherally evoked slow wave, and some relevant facts about the lemniscal and non-lemniscal pathways ascending to the cerebral cortex.

### KCs and slow waves

**Figure 2:**
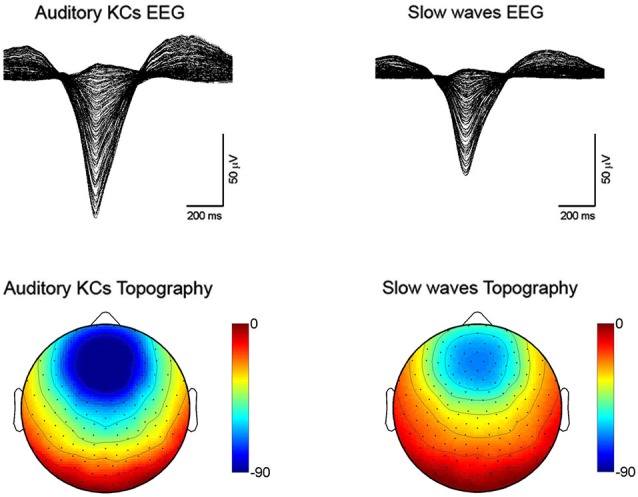
**Sleep recordings performed with a HydroCel 256 channel hdEEG net (Electrical Geodesics Inc.) using NetStation software in healthy subjects (*n* = 5)**. Auditory tones were delivered through speakers or headphones during N2-N3 sleep stages. Auditory KCs and slow waves were detected globally based on period-amplitude criteria (peak to peak minimum amplitude: 75 µV; negative going zero-crossing to positive going zero-crossings greater than 250 ms and less than 1000 ms). For the KCs, an additional criterion was that the waves had to occur between 200 and 1100 ms after the auditory stimulus. Top figures are butterfly plots (all channels overlaid) averaging across all auditory evoked KCs (on the left) and all spontaneous slow waves (on the right). Both waves were aligned by the negative peak. Bottom plots show the average scalp voltage topography at the negative peak for KCs (on the left) and spontaneous slow waves (on the right).

The KC was first described by Loomis in 1937 as a distinctive, large wave occurring during light stages of NREM sleep, often in response to sensory stimuli, and consisting of a brief negative peak of several hundreds microvolts followed by a slower positive component (Loomis et al., 1938). According to this definition, both KCs and spontaneous slow waves are “delta” waves occurring during NREM sleep (Colrain, 2005). Indeed, they both are characterized by a large and sharp negative deflection of the EEG line (Figure 2, top panels). Moreover, the topographic distribution of this negative component, which represents the biggest portion of the wave, is fronto-central and bilaterally symmetrical for both KCs and slow waves (Figure 2, bottom panels).

In addition, there is some evidence suggesting that KCs, like slow waves, might be homeostatically regulated. It has been reported that: (1) KCs density declines from evening to morning and from cycle to cycle across the night of sleep (De Gennaro et al., 2000; Curcio et al., 2003; Sforza et al., 2004; Halász, 2005); (2) KCs are more frequent during a night of recovery sleep following fragmented sleep compared to a baseline night (Nicholas et al., 2002b); (3) KC amplitude tends to be bigger after sleep deprivation, although results are inconsistent (Nicholas et al., 2002b; Peszka and Harsh, 2002; Curcio et al., 2003; Sforza et al., 2004); (4) KCs are smaller and rarer in the elderly and in alcoholics, who also show a large decline of SWA (Nicholas et al., 2002a).

A definitive link between slow waves and KCs was provided by Amzica and Steriade in experiments conducted in cats, which revealed the existence of a slow, cortically-generated oscillation within the thalamo-cortical system (Steriade et al., 1991, 1993b, 2001; Amzica and Steriade, 1998a). The slow oscillation is the fundamental cellular phenomenon that underlies both KCs and slow waves, and manifests as a bistability of the resting membrane potential, which transitions from a depolarized up state, when neurons show sustained firing, to a hyperpolarized down state, characterized by neuronal silence (Steriade et al., 1993b, 2001; Amzica and Steriade, 1998b; Destexhe et al., 1999). The mechanisms that trigger and terminate up and down states remain unclear, but it is known that depolarization-dependent K+ currents play a major role (McCormick et al., 1993; Steriade et al., 1993a; Sanchez-Vives and Mccormick, 2000; Timofeev et al., 2000b; Hill and Tononi, 2005), and it is now clear that layer V pyramidal neurons are especially critical for the regulation of up and down state dynamics (Beltramo et al., 2013). There is a close temporal relationship between these cellular phenomena and simultaneously recorded slow waves or KCs: the surface negativity in the EEG signal (or depth positivity in the local field potential, LFP) corresponds to the down state of cortical neurons as recorded intracellularly, and to the suppression of spiking activity as recorded extracellularly, suggesting that EEG or LFP slow waves are a reflection of near-synchronous transitions between up and down states in large populations of cortical neurons (Murata and Kameda, 1963; Calvet et al., 1973; Noda and Adey, 1973; Burns et al., 1979; Steriade et al., 1993c, 2001; Contreras and Steriade, 1995; Mölle et al., 2006; Mukovski et al., 2006; Ji and Wilson, 2007; Luczak et al., 2007; Cash et al., 2009). Therefore, the propensity of neurons to fall inevitably into silent, hyperpolarized states after a period of activation (bistability) underlies the occurrence of both KCs and slow waves during NREM sleep. However, what mechanisms lead specifically to the triggering of KCs or slow waves is still a matter of investigation.

Slow waves have classically been considered spontaneous events, whereas KCs can be triggered by sensory stimulation, most effectively by acoustic stimuli, but also other stimuli, including visceral ones (Davis et al., 1939; Ackner and Pampiglione, 1957; Webster and Colrain, 1998, for a review: Colrain, 2005). However, even with the most efficacious stimulation, not every peripheral stimulus can evoke a KC during NREM sleep. Some authors have suggested that the cerebral cortex may not always be equally receptive during NREM sleep (Prince, 1965; Terzano et al., 1985; Achermann and Borbély, 1997; Massimini et al., 2003; Vanhatalo et al., 2004; Parrino et al., 2012; Schabus et al., 2012). For example, Vanhatalo et al. (2004) observed a cyclic modulation (at 0.02–0.2 Hz) of cortical excitability in full-band EEG recordings during NREM sleep, reporting a highly synchronized occurrence of KCs with the negative deflection of this infra-slow fluctuation. Earlier animal studies showed that changes in cortical excitability could reflect rhythmic fluctuations of neuronal activity in the midbrain reticular formation (MRF; Roldán and Radil-Weiss, 1970; Oakson and Steriade, 1982, 1983), whereas more recent evidence suggests that these fluctuations can arise from an intrinsic instability of cortical neuronal networks (Sanchez-Vives and Mccormick, 2000; Timofeev et al., 2000a). Another well-known feature is that evoking two KCs in a short interval is virtually impossible because of a refractory period occurring after each KC (Firth, 1973; Bastien and Campbell, 1994). This feature has led to the suggestion that KCs may exert a protective role for cortical arousals (Colrain, 2005; Halász, 2005); specifically, the occurrence of KCs in relation to a potentially arousing stimulus could briefly reduce cortical excitability, the influence of sensory inputs and, thus, preserve sleep continuity.

Among different sensory modalities, auditory stimulation is the most reliable way to induce KCs in humans (Colrain, 2005; Halász, 2005; Riedner et al., 2011). The reason why the thalamo-cortical system should be particularly sensitive to acoustic stimuli during sleep remains unclear. Since humans are highly visual, one would expect visual stimuli to be at least as effective as acoustic stimuli in inducing KCs. However, unless particularly strong visual stimuli are used (Riedner et al., 2011) this seems not to be the case, probably because acoustic stimuli have a stronger influence on the arousal systems, given the intricate pattern of connections between the acoustic pathways and the reticular formation in the brainstem (Reese et al., 1995a; Yeomans and Frankland, 1995; Cant and Benson, 2003; Hu, 2003). Moreover, in micro-osmatic animals like humans, the auditory system may be the best system capable of monitoring the environment and detecting distant threats during sleep (Velluti, 1997). By contrast, in osmatic animals, like rodents, this sentinel role may be also played by the olfactory system, which may explain why olfactory stimuli are particularly effective in inducing slow waves in rodents (Fontanini et al., 2003; Fontanini and Bower, 2006).

**Figure 3:**
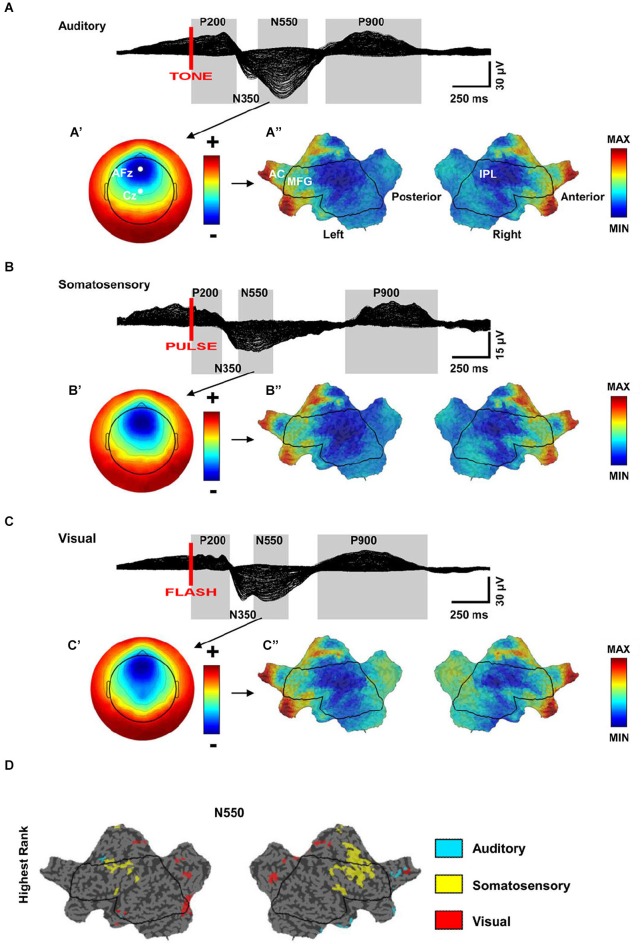
**(A–C)** Similarity of scalp and source topographies of K-complex (KC) responses. Across subject (*n* = 7) grand average 256-channel EEG butterfly plot (overlaid traces) of the evoked response during sleep for auditory, somatosensory, and visual stimulation. **(A–C’)** Scalp topography for the N550 time periods. Each map is independently scaled in order to indicate relative topography. Red indicates positivity with respect to the average and blue indicates negativity. **(A’)** ranges from −30 (blue) to +20 (red); **(B’)** ranges from −10 (blue) to +7 (red); **(C’)** ranges from −23 (blue) to + 17 (red). **(A–C”)** Flat maps of the cortical sources for the N550 peak. Current hot spots (most current) indicated in red, cold spots in blue. AC = Anterior Cingulate, MFG = Middle Frontal Gyrus, IPL = Inferior Parietal Lobule. **(ABC”)**, respectively MIN = −1.3, −1.2, −1.4; MAX = 2.6,2.5,2.3. **(D)** Modality-specific differences in cortical sources for the N550 peak of KC. Flat map of significantly different cortical sources across stimulation modalities (Quade test, *p* < 0.05). Color-coding of voxels indicates the stimulation with the highest ranking relative to the other stimulation modalities (adapted from Riedner et al., 2011).

K-complexes are highly stereotypical in shape and topographic distribution, independent of the stimulus used to evoke them. Because of this consistency, they are often viewed as the result of a non-specific diffuse response of the brain to any sensory stimulus (Numminen et al., 1996; Halász, 2005; Riedner et al., 2011). However, this notion has been challenged by recent studies that have provided evidence for the activation of specific primary sensory areas during the induction of KCs, suggesting the existence of a local component in conjunction with the more diffuse activation. A recent study performed source modeling of high-density EEG recordings in NREM sleep during three different kinds of stimulation (auditory, somatosensory, and visual) (Riedner et al., 2011). As expected, similar source topography was observed for the large negative portion of the KC across different stimulations, although the magnitude of the activation appeared largest for the acoustic stimulation. A more fine-grained analysis, however, showed that some aspects of the KC response were not entirely sensory pathway independent (Figure 3). Specifically, higher activation of primary visual areas was observed after visual stimulation, while primary somatosensory and motor areas were more activated after somatosensory stimulation. The primary auditory area, instead, showed a similar level of activation during all types of stimulation, including acoustic stimulation. The authors suggested that the lack of a specific relative auditory activation could have been related to the fact that auditory cortex is particularly difficult to image with source modeling, but could also have been related to the fact that this area may be involved in all evoked KCs, regardless of stimulation modality (see below) (Riedner et al., 2011). However, neuroimaging studies have found that during the presentation of the subject’s name or following an acoustic tone the auditory cortex is activated to the same extent in wake and NREM sleep (Portas et al., 2000). Another study that combined EEG and fMRI during NREM sleep found that only tones that were able to evoke a KC led to an activation of primary auditory cortex (Czisch et al., 2009; Dang-Vu et al., 2011). Of note, the same study also showed concomitant activation of the frontal midline regions during the tone-evoked KC (Czisch et al., 2009).

Thus, these results confirm that KCs are characterized by (1) a diffuse brain response that is topographically consistent across different sensory stimulation modalities, but more pronounced for tones; and (2) a local response that is sensory specific and involves primary sensory areas. How sensory stimulation can elicit this dual response is still unclear. One possibility is that the diffuse response is induced by an initial activation of the primary sensory areas that expands through cortico-cortical connections. An alternative explanation is that local and diffuse components reflect the parallel activation of specific and nonspecific ascending sensory pathways, respectively.

## Lemniscal and non-lemniscal ascending pathways

Ascending sensory pathways are anatomically subdivided into two systems across almost every sensory modality: primary (lemniscal) and secondary (non-lemniscal) (Hu, 2003). Lemniscal pathways are reported to carry a high-fidelity, primary-like representation of stimulus features, while the non-lemniscal pathways supply more information about environmental changes (Kraus et al., 1994; Anderson et al., 2009; Anderson and Linden, 2011). Moreover, the non-lemniscal pathways are usually sensitive to multimodal stimuli (Komura et al., 2005) and display rapid habituation to repetitive stimulation (Calford and Aitkin, 1983; Edeline et al., 1999; Hu, 2003).

In the auditory system, the lemniscal pathways transmit auditory inputs ascending from the cochlear nuclei, while the non-lemniscal pathways consist of fibers coming from several regions of the brainstem. These include the dorsal nucleus of the inferior colliculus (IC), the MRF, the nucleus sagulum (Sag), and the spinothalamic tract (ST; Hu, 2003). In addition, cholinergic nuclei of the pontine reticular formation (PRF) that receive direct afferents from cochlear nuclei send their contribution to non-lemniscal pathways (Mesulam et al., 1983; Steriade, 1990; Reese et al., 1995b).

In the auditory thalamus (medial geniculate body, MGB) lemniscal and non-lemniscal pathways are clearly segregated and target different neuronal populations. According to Jones’s (1998, 2001) hypothesis, thalamic relay cells can be divided in two classes: core and matrix. Core cells receive lemniscal inputs and transmit to primary sensory cortex (layer IV). Matrix cells are targeted by non-lemniscal fibers and diffusely project to associative cortical areas, mainly to layer I. This hypothesis holds for the auditory thalamus (Jones, 1998, 2001; Clascá et al., 2012), where core cells are abundant in the ventral portion of MGB (MGv) that receives lemniscal inputs, whereas matrix cells, targeted by non-lemniscal fibers, prevail in the dorsal and caudo-medial MGB (MGd/MGm) (Jones, 1998; Clascá et al., 2012). It has been proposed that these two regions engage different aspects of auditory function (Hu, 2003). The MGv is tonotopically organized and transmits high-fidelity information to the cortex representing the details of the acoustic environment. The MGd/MGm, instead, projects broadly to the upper layers of associative areas surrounding the primary auditory region, favoring the integration across modalities and across frequencies, displaying delayed responses to acoustic stimuli, and showing rapid habituation to unvarying stimuli (Calford and Aitkin, 1983; King et al., 1995; Miller et al., 2001a,b; He and Hu, 2002; Hu, 2003; Lee and Sherman, 2011). In addition, MGd/MGm seems to have a unique ability to activate the cortex when changes are detected in an otherwise monotonous series of acoustic stimuli (Kraus et al., 1994).

**Figure 4:**
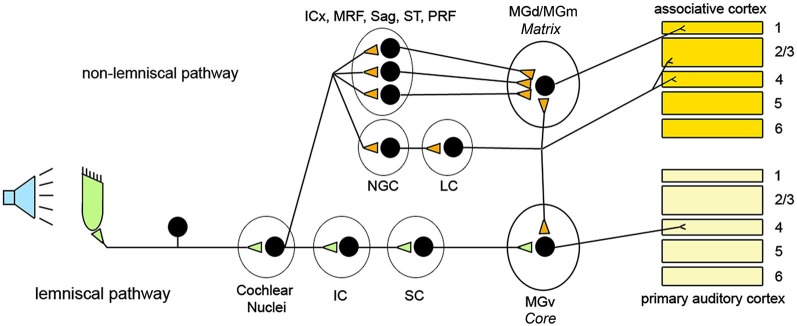
**Schematic representation of the organization of the ascending acoustic pathways and relative targets in the thalamus and the cerebral cortex**. ICx: shell of the inferior colliculus; MRF: midbrain reticular formation; Sag: Sagulum; ST: spinothalamic tract; PRF: pontine reticular formation; NGC: Nucleus gigantocellularis; LC: Locus coeruleus; IC: Inferior colliculus; SC; Superior colliculus; MGd: Medial geniculate dorsal; MGm: Medial geniculate caudo-medial; MGv: Medial geniculate ventral.

The nucleus gigantocellularis (NGC), in the upper part of the medullary reticular formation, also receives auditory inputs (Martin et al., 2010; Pfaff et al., 2012). This nucleus is connected with several brainstem structures, and plays a major role in activating neurons of the locus coeruleus (LC), the major source of noradrenaline in the brain (Aston-Jones et al., 1986; Van Bockstaele and Aston-Jones, 1995; Berridge and Waterhouse, 2003). In contrast to brainstem glutamatergic and cholinergic ascending projections, which mainly innervate sub-cortical structures, LC neurons innervate directly all cortical layers (Jones and Moore, 1977; Jones et al., 1977; Jones and Yang, 1985; Jones, 2003), fire maximally in response to novel stimuli (Aston-Jones et al., 1996), and are highly effective in activating the cerebral cortex (Berridge and Foote, 1991; Carter et al., 2010; Constantinople and Bruno, 2011). Interestingly, LC fibers and other ascending fibers of the non-lemniscal pathways show rapid habituation to repeated presentation of the same acoustic stimulus (Hervé-Minvielle and Sara, 1995). Overall, there is substantial anatomical overlap between the auditory non-lemniscal pathways and the arousal promoting systems that diffusely project to the thalamo-cortical system, including LC fibers and pontine cholinergic projections (Hu, 2003; Jones, 2003). Since these arousal systems can be broadly activated by acoustic stimulation, we include their ascending projections as part of the “non-lemniscal” pathways (Figure 4).

## Slow wave enhancement: a hypothetical mechanism

We have seen that non-lemniscal pathways include arousal-promoting neuromodulatory systems with diffuse thalamic and/or cortical projections, but also thalamic matrix cells, which have diffuse cortical projections as well, especially to layer I (Jones, 2001). While the involvement of these projections in activating the thalamo-cortical system during sleep is well established (Jones, 2003; Halász et al., 2004; Riedner et al., 2011), less is known about their role in triggering slow waves in response to a sensory stimulus.

We hypothesize that during NREM sleep auditory inputs capable of activating non-lemniscal pathways can produce near-simultaneous depolarization of many neurons widely distributed over the cortex. Provided that it does not arouse the subject, such “bottom-up” activation may lead to a fast and efficient synchronization of large populations of cortical neurons. Given the bistable behavior of the thalamo-cortical system at this stage of sleep, the rapid and synchronous neuronal depolarization would be inevitably followed by a massive hyperpolarization. At the EEG level, this would lead to an “enhanced” slow wave that displays larger amplitude, steeper slope, and involves broader cortical regions than the majority of spontaneous slow waves, which typically originate independently in many regions of the cortex and involve circumscribed cortical areas (Nir et al., 2011; Siclari et al., 2014). Thus, the net result of the emergence of acoustically induced slow waves during NREM sleep would be an increase in SWA, in line with what has been observed experimentally in several studies (Tononi et al., 2010; Arzi et al., 2012; Ngo et al., 2013a,b, see also Figure 1).

## Slow wave induction and arousals

**Figure 5:**
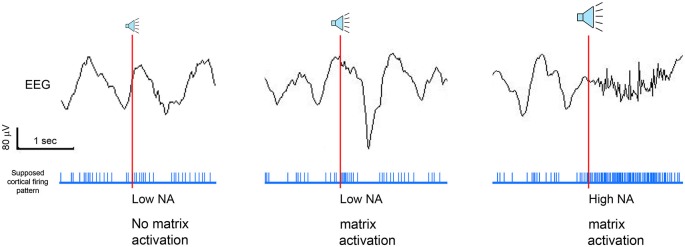
**Schematic representation of the hypothetical role of thalamic matrix cells and noradrenaline (NA) in regulating the EEG outcome after tone presentation**. We hypothesize that, during NREM sleep, acoustic stimuli can be ineffective, lead to enhanced waves, or provoke cortical arousals, depending on the involvement of the locus coeruleus and/or thalamic matrix cells.

We have seen that the mechanism by which slow waves can be induced is likely the same one used to arouse the organism when a sudden change indicating a potential danger in the environment is detected. Thus, the intensity of stimulation has to be strong enough to trigger the ascending pathways, but not so strong as to cause a full-blown awakening. This suggests the existence of a threshold below which the stimulation is likely to be completely ineffective, above which the stimulation will be effective, and further above which the stimulation is likely to be disruptive. The idea that the arousal systems can be functionally parceled according to the magnitude of stimulation is not new, and relies on an old concept introduced by Moruzzi in early 1950s. Specifically, he claimed that for mild sensory stimulation only some portions of the activating reticular ascending system (ARAS) might be activated, while the entire system could be recruited only by more intense stimuli (Moruzzi, 1954; Berlucchi, 1997). Although still lacking direct confirmation, there is experimental evidence demonstrating that not all parts of the ARAS are equally effective in arousing the thalamo-cortical system. For example, Constantinople and Bruno (2011) observed that the activation of the noradrenergic, but not cholinergic, pathways were capable of changing the cerebral cortex from a state of slow wave anesthesia to wake. The key role of LC in promoting waking has been confirmed in optogenetic experiments that targeted one arousal system at a time. In these studies, only the activation of LC caused almost immediate sleep-to-wake transitions from both NREM or REM sleep, suggesting that the LC, more than other arousal pathways, is crucial in promoting wakefulness (Carter et al., 2010; de Lecea et al., 2012). Other systems instead, may either take longer to wake up the subject (e.g., the orexinergic system; Adamantidis et al., 2007), or may be equally effective in promoting the transition from NREM sleep to REM sleep or wake (Han et al., 2014) Therefore, it is reasonable to hypothesize that low-intensity stimuli that induce slow wave enhancement are strong enough to activate several parts of the ARAS, but not the LC (Figure 5).

## Optimization of acoustic stimulation for slow wave enhancement during SWS

Having developed a framework for understanding how acoustic stimulation enhances slow waves during sleep, we now consider which features of the stimulation might be most impactful when considering these mechanisms. By reviewing the relevant literature, we point out several features of the acoustic stimulation that may affect the magnitude of the slow wave enhancement. By optimizing these parameters, it should be possible to maximize the effectiveness of the stimulation for increasing slow waves.

1. *Intensity*. The intensity of the acoustic stimulation should be adjusted according to sleep depth in order to avoid undesired arousals and sleep fragmentation. We have previously discussed the existence of a threshold below which the stimulation intensity is effective in enhancing slow waves and above which it causes arousal, and how the activation of the LC can represent the key factor in tuning the balance. However, it is still not clear how this threshold can be defined dynamically throughout the course of sleep and whether there are some features of the EEG signal that can be used to predict it and adjust the stimulation accordingly. A recent study showed that subjects displaying high spindle density during NREM sleep were more resistant to sleep disruption during auditory stimulation, suggesting that spindle density can be a good indicator of sleep stability (Dang-Vu et al., 2010). Other studies observed that the intrinsic frequency of the spindle changes within a sleep cycle, especially for more frontal spindles (Himanen et al., 2002; Andrillon et al., 2011). Specifically, the frequency of the spindles decreases as sleep deepens and it increases as sleep lightens, describing a U-shape curve within the sleep cycle (Himanen et al., 2002; Andrillon et al., 2011). Since the level of thalamic hyperpolarization dictates the period of spindle oscillations (McCormick and Bal, 1997), it is possible that the frequency of the spindles reflects the level of thalamo-cortical polarization (Andrillon et al., 2011). Thus, the deceleration of spindle frequency reported in deep sleep may indicate an increased hyperpolarization of the thalamo-cortical system during this stage, leading to a stronger resilience to perturbing stimuli. Taken together, these studies suggest that measuring spindle density and frequency can provide important information about the state of the thalamo-cortical system and its sensitivity to external stimuli, and therefore can be used to determine the intensity of the stimulation at any given time during NREM sleep.
2. *Sound frequency*. The sound frequency of the acoustic stimuli should vary randomly for each stimulus to prevent habituation of the non-lemniscal ascending pathways. In contrast to the lemniscal pathways that constantly exhibit high-fidelity responses, non-lemniscal pathways show rapid habituation to trains of identical stimuli (Calford and Aitkin, 1983; King et al., 1995; Miller et al., 2001a,b; He and Hu, 2002; Hu, 2003; Lee and Sherman, 2011). Since the early studies performed in late 1950’s by Sokolov (1963) habituation has been characterized as a decreasing pattern of response following repeated sensory stimulation. It was originally thought that habituation was caused by a blockade of inputs to the reticular formation exerted by corticofugal projections (Sokolov, 1963). However, subsequent studies showed that the mechanism underlying habituation resides in the reticular formation itself and is due to a progressive reduction of synaptic efficacy (Groves and Lynch, 1972; Weber et al., 2002; Thompson, 2009). If the function of non-lemniscal pathways is to detect sudden unexpected changes in the environment, then habituation becomes an essential feature to maximize the capability of an organism to sense change in a dynamic environment, and, conversely, to ignore events displaying a repetitive pattern that contain little new useful information. Therefore, when attempting to enhance slow waves it is essential to provide a certain continuous degree of unexpectedness during stimulation, in order to avoid habituation. One possible way to achieve this is suggested by the evidence that, in a sequence of acoustic stimuli, deviant tones showing a sound frequency different from the background elicit an EEG event-related potential called the mismatch negativity in awake subjects (Näätänen et al., 2011). This characteristic response is thought to reflect the brain reaction to changes occurring in a continually updated auditory environment (Winkler et al., 1996; Sussman and Winkler, 2001), and, albeit attenuated in NREM sleep (Chennu and Bekinschtein, 2012), it can be used to diminish habituation and maintain the desirable effect. In practice, a feasible approach would consist of changing sound frequency of every acoustic stimulus in a sequence of stimuli.
3. *Timing*. Ideally, the stimulation should arrive to the cortex during the particular phase of the thalamo-cortical oscillation in which its ability to enhance slow waves is maximal. During SWS, slow waves occur more or less regularly one after another, suggesting that the underlying thalamo-cortical network swings continuously between up and down states. Experimental evidence suggests that the phase of this ongoing oscillation has important consequences for the fate of incoming stimuli. For example, the amplitude of somatosensory evoked potentials tends to increase progressively when the stimulus occurs during the negative slope of the wave (i.e., when thalamo-cortical neurons are becoming hyperpolarized), reaches its maximum at the negative-positive transition, and decays along the positive drift (Massimini et al., 2003). On the other hand, the amplitude of auditory evoked potentials and BOLD responses in the superior temporal gyrus, a higher auditory association cortex, is larger when stimulation occurs just after the negative peak of the EEG slow wave (i.e., when thalamo-cortical neurons are depolarizing) (Schabus et al., 2012). It is hard to evaluate whether either different delays of somatosensory and auditory systems, or inherent delays in the stimulating systems might be responsible for these different results. However, a recent study, using a precise stimulating apparatus with an inherent delay of a few milliseconds, demonstrated that acoustic pulses occurring in correspondence of the slow wave up states were effective in enhancing subsequent slow waves. Conversely, tones delivered immediately after the negative peak of the slow waves had a disruptive effect on the following waves, most likely by interfering with the intrinsic bistable activity of the thalamo-cortical system (Ngo et al., 2013b). These findings are consistent with a previous study performed in rats showing the presence of a refractory period right after the spontaneous EEG down state, when the induction of slow waves was unlikely even under the highest physiological pressure for sleep (Vyazovskiy et al., 2009). Overall, these data indicate that the effect of incoming stimuli is strongly influenced by the phase of the thalamo-cortical oscillation. Thus, the ability of acoustic stimuli to trigger a slow wave relies not only on sufficient activation of the ascending sensory pathways, but is also strongly dependent on the phase of the ongoing oscillation of the thalamo-cortical system.
4. *Entrainment*. In addition to maximizing the efficacy of the response to an individual stimulus, slow wave enhancement can take advantage of the brain’s ability to entrain to repetitive stimuli. Entrainment refers to the ability of a periodic external force to synchronize the natural oscillation of a certain system (Pikovsky et al., 2003). Entrainment of EEG brain waves is a well-known phenomenon (Herrmann, 2001; Thut et al., 2011). The photic driving response was discovered in 1934 by Adrian and Matthews, who first demonstrated the entrainment of the EEG alpha rhythm using intermittent photic stimulation (Adrian and Matthews, 1934). Since then, this technique has been widely used to induce abnormal EEG features in an otherwise normal EEG (Takahashi, 2005). The photic driving response is usually elicited when the stimulation frequency is similar to the intrinsic frequency of the brain oscillations, and it is thought to represent a steady-state resonance response of the brain to periodic stimulations (Walter and Walter, 1949; Mundy-Castle, 1953). Electroencephalogram entrainment to rhythmic sensory stimulation is not limited to the visual modality, and has been reported after somatosensory and auditory stimulation (Pompeiano and Swett, 1962; Rodenburg et al., 1972; Sclabassi et al., 1974). Specifically, slow oscillations can be entrained by rhythmic auditory stimulation during SWS in animals and humans (Gao et al., 2009; Ngo et al., 2013a). The underlying mechanism is not well elucidated, but the intrinsic pacemaker activity of neurons and synaptic connections may give rise to entrainable oscillators (Gao et al., 2009). If so, auditory stimulation could act as a driving force able to organize and synchronize intrinsic brain oscillators. At the EEG level, this would result in waves more organized around the frequency of stimulation, and in increased power associated with that frequency (Ngo et al., 2013a). This view is also in line with the increase of slow oscillation activity (EEG power between 0.5 and 1 Hz) during acoustic stimulation at 0.8 Hz, roughly the spontaneous frequency of the thalamo-cortical system during SWS (Ngo et al., 2013a).

## Closed-loop stimulation

We have seen that slow wave enhancement can be optimized by tuning some features of the acoustic stimulation. However, a limiting factor in the ability of enhancing slow waves is that the mechanism responsible for this effect is also the same one used to arouse the cortex from sleep. Playing tones at inappropriate volumes or at inappropriate times during sleep not only can reduce the desirable effect, but can induce arousals, and disrupt sleep. It becomes then imperative to adjust the stimulation parameters moment by moment according to the depth of sleep and, as described above, the brain’s changing receptiveness to incoming stimuli.

**Figure 6:**
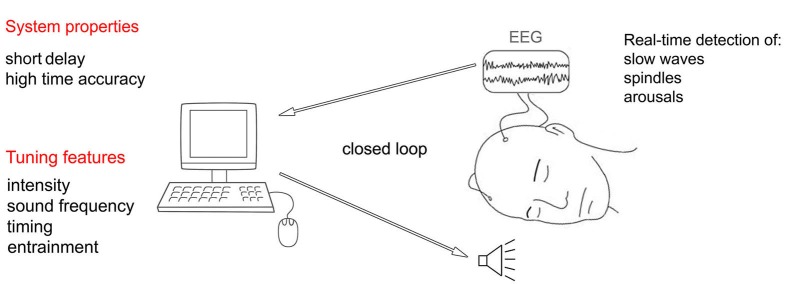
**Schematic representation of an automated real-time system capable of adjusting acoustic stimulation parameters according to the ongoing sleep**.

In practice, such dynamic control can be achieved by developing a system that monitors the EEG and, in real-time, assesses first whether the stimulation can be delivered without the risk of arousing the user, and then adjusts the stimulation properties depending on the ongoing EEG (Figure 6). From an implementation perspective the system estimates, with a high sensitivity, the likelihood of an arousal event by considering the EEG power in the higher frequency bands (alpha and beta). In case an arousal is detected during stimulation, the latter should cease. If a sufficiently long period of time (few minutes) has passed since the last arousal event, then the system should attempt to detect (with high specificity) the occurrence of SWS. In case SWS is detected, then the acoustic stimulation should be delivered and its properties adjusted to the ongoing EEG. Specifically, if the slow wave enhancement effect decreases, the intensity of the stimulation increases.

Crucial properties of such real-time system include small inherent delay in acquiring EEG data and high timing accuracy (low jitter) in delivering the stimuli (Hartmann et al., 2011). For example, short delay and narrow jitter would make it possible to target a precise phase of the on-going oscillation or would allow the system to quickly reduce the stimulation intensity when high frequency EEG patterns first appear, heralding an arousal.

## Acoustic stimulation outside NREM sleep

There is some evidence that acoustic stimulation could be used not only to enhance NREM slow waves, but also some features of REM sleep (Drucker-Colin et al., 1983; Arankowsky-Sandoval et al., 1986, 1992; Vazquez et al., 1998; Amici et al., 2000). Early studies found an increased number of ponto-geniculo-occipital (PGO) spikes and increased REM duration in animals exposed to auditory stimulation. The authors attributed this effect to the anatomical proximity of the structures involved in acoustic signal processing and REM sleep regulation. More specifically, they suggested that acoustic stimulation could promote the release of acetylcholine in the brainstem structures involved in PGO activity (Arankowsky-Sandoval et al., 1986; Ball et al., 1991). The increased REM duration following acoustic stimulation was later confirmed by another group of researchers showing longer REM periods also for stimulations occurring during NREM sleep (Amici et al., 2000). In humans, an increase in both REM sleep duration and sleep efficiency occurs when acoustic stimulation is started at the beginning of REM sleep, whereas a disruptive effect with a larger number of awakenings has been reported when the stimulation starts near the end of a REM episode (Salin-Pascual et al., 1991). Increased REM sleep duration was also correlated with a pronounced decrease in the density of REMs (Mouze-Amady et al., 1986), and to a better retention of memories in a Morse code learning task (Guerrien et al., 1989). Nevertheless, despite several reports indicating that acoustic stimulation lengthens REM sleep, the behavioral impact of this manipulation requires further investigation.

## Summary and future directions

Slow waves are the best marker of the homeostatic regulation of sleep, and, most likely, they are responsible for carrying out some physiological functions of sleep for the brain (Tononi and Cirelli, 2003, 2006, 2014). Novel data indicate that it is possible to enhance these slow waves through non-pharmacological means. Initial experiments showed that tDCS and TMS applied to the human cerebral cortex at appropriate frequencies could induce slow waves (Marshall et al., 2006; Massimini et al., 2007). However, these methods are currently impractical and their safety, especially for chronic long-term exposure, is still unknown. Recently, more attention has been given to the possibility of enhancing slow waves by using more physiological stimuli. Among different sensory modalities, acoustic stimulation appears to be the most effective in increasing the magnitude of slow waves. The underlying mechanism is unclear, but we hypothesize that sub-arousal threshold stimuli are capable of synchronizing the cortical activity of large populations of neurons through the activation of the non-lemniscal pathways that project diffusely over the cerebral cortex. Given the bistable behavior of the thalamo-cortical system during NREM sleep, the rapid and synchronous neuronal depolarization would be inevitably followed by a massive hyperpolarization. At the EEG level, this would result in a slow wave showing large amplitude and steep slope, and involving bilaterally the fronto-central regions.

We then considered which features of the stimulation might be most impactful when considering these mechanisms. By reviewing the relevant literature, we pointed out several features (intensity, sound frequency, timing, and entrainment) of the acoustic stimulation that may play an important role in regulating the efficacy of the stimulation. Specifically, we indicate that stimulation intensity should be tuned according to sleep depth, because there is a threshold below which the stimulation intensity is effective in enhancing slow waves and above which it causes arousal. Changing the sound frequency could counteract the occurrence of habituation and hitting the thalamo-cortical system at convenient times could maximize the enhancing effect. Finally, we argue that repetitive patterns of stimulation can easily entrain endogenous brain rhythms leading to waves more organized around the frequency of stimulation. However, it remains to be determined to what extent these features can be tuned and how they relate to each other. For example, it is possible that hitting the precise phase of the ongoing thalamo-cortical oscillation becomes less important when the brain is entrained by a repetitive stimulation. Moreover, the interaction of these factors may be different in specific subsets of the population, for instance in subjects who are easily aroused during sleep by environmental noise, or in elderly people who typically exhibit reduced amount of SWA. Finally, it would be interesting to assess the potential role of acoustic stimulation during sleep in conditions characterized by decreased SWS, such as chronic sleep restriction and insomnia.

## Conflict of interest statement

Dr. Tononi has a consulting agreement with Philips Respironics. He is also the David P. White Chair in Sleep Medicine, an endowed chair to the University of Wisconsin made available by a contribution from Philips Respironics. Dr. Garcia-Molina is salaried employee of Philips. In addition, the authors are listed on a number of pending patent applications related to this work.
